# Clinical Features and Prospective Outcomes of Thin-Filament Hypertrophic Cardiomyopathy: Intrinsic Data and Comparative Insights from Other Cohorts

**DOI:** 10.3390/jcm14030866

**Published:** 2025-01-28

**Authors:** Olga S. Chumakova, Tatiana N. Baklanova, Dmitry A. Zateyshchikov

**Affiliations:** 1 Moscow Healthcare Department, City Clinical Hospital 17, 119620 Moscow, Russiazateyschikovda@zdrav.mos.ru (D.A.Z.); 2 E.I. Chazov National Medical Research Center for Cardiology, 121552 Moscow, Russia

**Keywords:** hypertrophic cardiomyopathy, thin filament, sarcomere, outcomes, *TNNI3*, *TNNT2*, *TNNC1*, *TPM1*, *ACTC1*

## Abstract

**Background/Objectives**: Hypertrophic cardiomyopathy (HCM) is the most common genetic heart disease. The most frequently mutated genes encode proteins of the thick filament of the sarcomere, while mutations in thin-filament genes are rare findings in HCM cohorts. Recent studies have revealed distinct mechanisms of disease development linked to thin-filament mutations, highlighting the need for further investigation into this rare subgroup. **Methods**: A total of 82 adult patients with sarcomere-positive HCM were enrolled. Baseline characteristics and nearly five years of follow-up data from 15 patients with thin-filament mutations were analyzed and compared with those from 67 patients with thick-filament mutations and findings from other studies. **Results**: Compared to thick-filament HCM patients, individuals with thin-filament mutations exhibited significantly lower maximum left ventricular wall thickness, as measured by both echocardiography (*p* = 0.024) and cardiac magnetic resonance (*p* = 0.006), showed more rapid progression to advanced heart failure (HR = 5.6, *p* = 0.018), and less often underwent septal reduction therapy (*p* = 0.025). None of the thin-filament HCM patients experienced malignant arrhythmic events. **Conclusions**: In adults, thin-filament HCM is associated with a ‘thinner’ phenotype and a more rapid progression to advanced heart failure compared to thick-filament HCM. Data on a higher risk of malignant arrhythmias in thin-filament HCM remain controversial between studies and rather depend on the age of onset and genotype in each particular family.

## 1. Introduction

Hypertrophic cardiomyopathy (HCM) is the most common genetic heart disease, characterized by left ventricle (LV) hypertrophy in the absence of abnormal loading conditions [1]. Its clinical course ranges from mildly symptomatic longevity to sudden cardiac death (SCD), advanced heart failure (HF), or embolic stroke.

Currently available data indicate that the genetic basis of HCM is complex and includes dozens of genes [2]. In approximately 50% of cases, HCM is caused by (likely) pathogenic variants (previously mutations) in genes encoding sarcomeric proteins. Sarcomeres, the basic contractile units of cardiomyocytes, facilitate contraction through cyclic interactions between the thick-filament protein myosin and thin-filament protein actin. This process is finely regulated by the troponin–tropomyosin complex, a component of the thin filament. Calcium binding to troponin triggers conformational changes in both troponin and tropomyosin which expose binding sites on actin, thereby enabling actin-myosin cross-bridge formation and subsequent muscle contraction [3].

The most frequently mutated genes in HCM (80% of genotype-positive cases) encode thick-filament proteins, including β-myosin heavy chain, *MYH7* (MIM #192600), and myosin-binding protein C, *MYBPC3* (MIM #115197) [4]. A smaller proportion of patients (an average of 6% from those submitted to genetic testing) carry (likely) pathogenic variants in thin-filament genes, such as alpha-actin, *ACTC1* (MIM #612098), troponin C, *TNNC1* (MIM #613243), troponin I, *TNNI3* (MIM #613690), troponin T, *TNNT2* (MIM #115195), and alpha-tropomyosin, *TPM1* (MIM #115196) [5].

Mutations in β-cardiac myosin lead to hypercontractility of the heart [6], a primary defect in HCM that precedes LV hypertrophy [7]. This knowledge has guided the development of cardiac myosin inhibitors, small molecules that reduce sarcomere contractility and mitigate HCM features [8,9]. While LV hypercontractility is a shared hallmark of HCM, the underlying mechanisms differ between thick- and thin-filament mutations [10]. Thick-filament HCM is primarily associated with increased ATPase activity and an elevated disordered relaxed state of myosin [6]. Conversely, thin-filament mutations initially disrupt calcium regulation: increased Ca^2+^ buffering and altered handling contribute to pathogenesis via Ca^2+^-dependent signaling pathways [11].

Some studies have described distinctive clinical presentations and outcomes in HCM patients with thin-filament mutations, in particular a milder phenotype accompanied by a high incidence of SCD [12,13,14]. However, these findings remain controversial [15,16,17], and further prospective research is needed to better understand this rare HCM subgroup.

This study aimed to evaluate the clinical features and prospective outcomes of patients with HCM caused by the causative variants in thin-filament sarcomeric genes, in comparison to those with thick-filament HCM. Additionally, results were interpreted in the context of previous studies.

## 2. Materials and Methods

Sarcomere-positive HCM patients from an ongoing single-center prospective study (protocol previously described in [18]) were enrolled. The diagnosis of HCM was established based on imaging criteria, defined as increased LV wall thickness ≥15 mm (≥13 mm for relatives) not solely explained by loading conditions. Cardiac imaging was performed using the Philips iE33 (The Netherlands) and GE HealthCare E90 (Chicago, IL, USA) echocardiograph systems by a single cardiomyopathy specialist, O.S.C. All eligible patients provided written informed consent, followed by clinical evaluation, including detailed personal and family history, and 12-lead electrocardiogram at rest. All patients were recommended for 24 h Holter monitoring and contrast-enhanced cardiac magnetic resonance (CMR) imaging if no previous data were available. Additionally, blood samples were collected for further genetic testing, which was performed in certified laboratories available at the time of patient enrollment. The pathogenicity of the identified variants, as determined by the laboratories, was evaluated in accordance with the 2015 joint consensus recommendations of the American College of Medical Genetics (ACMG) and Genomics and the Association for Molecular Pathology [19]. Furthermore, the authors reassessed the variants using predictive tools with integrated ACMG classification criteria and internal database analyses at the time of manuscript preparation. The study included carriers of single (likely) pathogenic variants or single variants of uncertain significance (VUSs) with potential disease-causing effects. Carriers of variants in *ACTC1*, *TNNC1*, *TNNI3*, *TNNT2*, and *TPM1* (thin-filament group) were compared with those harboring variants in *MYBPC3*, *MYH7*, *MYL2*, and *MYL3* (thick-filament group) regarding baseline characteristics, treatment approaches, and clinical outcomes. The outcomes analyzed were the following: (1) all-cause mortality; (2) malignant arrhythmic events, including SCD, successful resuscitation, and appropriate implantable cardioverter–defibrillator (ICD) shocks; (3) stroke (fatal and non-fatal); and (4) HF outcomes, including new HF progression and death from HF; HF progression was defined as hospitalizations requiring parenteral infusion of diuretics and/or inotropes, transition to hypokinetic HCM with a decrease in LV ejection fraction below 50%, or first occurrence of New York Heart Association (NYHA) class III/IV. The frequency of ICD insertion and septal reduction therapy was assessed over a single time period, including past history and follow-up. Additionally, the thin-filament group was evaluated in the context of previously reported data on thin-filament HCM from the literature.

### Statistical Analysis

Continuous variables are presented as the mean ± standard deviation or as the median with interquartile range (the difference between 75th and 25th percentiles) for non-normally distributed data. Comparisons were made using Student’s *t*-test or the Mann–Whitney U-test, as appropriate. Categorical variables are reported as counts and percentages and were analyzed using the chi-square test or Fisher’s exact test. Survival analyses were conducted using Cox proportional hazard regression. Survival curves and times were generated using the Kaplan–Meier method and compared with the log-rank test. All *p*-values were two-tailed and considered significant at <0.05. Statistical analyses were performed using SPSS version 26.0.

## 3. Results

Of the 230 genotyped HCM patients, 88 (39%) were sarcomere-positive. Six patients with multiple (likely) pathogenic variants were excluded. Of the remaining 82 patients with single sarcomeric variants, fifteen (18%) carried nine unique variants in thin-filament genes (one in *ACTC1*, two in *TNNI3*, two in *TNNT2*, one in *TNNC1*, and nine in *TPM1*) and sixty-seven (82%) carried forty-five unique variants in thick-filament genes (forty-one in *MYBPC3*, twenty-three in *MYH7*, and three in *MYL2*) (Supplementary Table S1).

### 3.1. Baseline Characteristics

Patients with thin-filament HCM (n = 15) were diagnosed and enrolled in the study at a median age of 44 and 49 years, respectively; probands comprised 80% of the group; half of the group presented with HCM-associated symptoms such as dyspnea (n = 7, 47%), palpitations (n = 4, 27%), and chest pain (n = 4, 27%); two (13%) had non-sustained ventricular tachycardia (NSVT) on Holter monitoring; of the probands (n = 12), seven had familial HCM, and two had SCD in families. All baseline characteristics, including imaging and electrocardiographic data, and comparisons between thin- and thick-filament HCM groups are shown in Table 1. Significant differences are bolded. All participants were Caucasian; 88% were of Eastern Slavic origin.

Patients with thin-filament HCM exhibited significantly lower maximal LV wall thickness values, as measured by both echocardiography (*p* = 0.024) and CMR (*p* = 0.006). They also had smaller indexed LV end-diastolic volumes on CMR (*p* = 0.041) and a lower 5-year SCD risk score (*p* = 0.007). Although patients in the thick-filament group were more frequently indicated for ICD insertion based on an SCD risk score > 6%, this difference did not reach statistical significance (*p* = 0.06). No other differences in baseline characteristics were observed.

### 3.2. Follow-Up and Treatment

**Figure 1:**
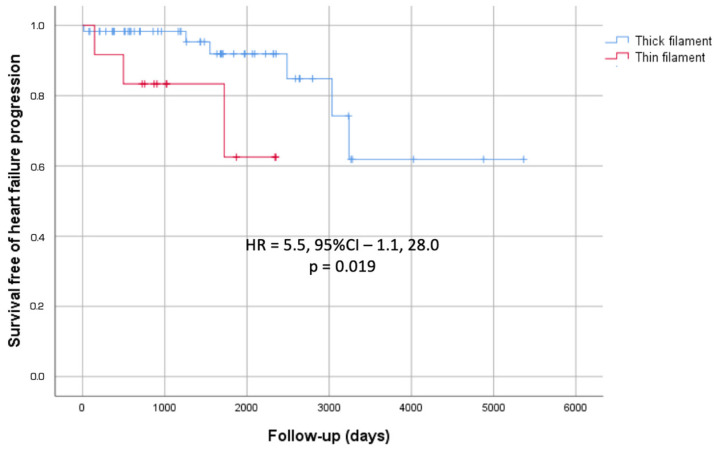
Kaplan–Meier cumulative incidence curves for HF outcomes in sarcomere-positive HCM probands (N = 69). Patients are stratified according to the presence of a causative variant in any sarcomeric thin-filament gene. The *Y*-axis represents proportions.

Follow-up duration was similar for thin- and thick-filament groups (4.7 ± 2.3 vs. 4.4 ± 3.4 years; *p* = 0.79). Of eighty-two patients, two died due to HF (both from the thick-filament group), one had a non-fatal stroke (from the thick-filament group), and nine progressed to severe HF (three from the thin-filament group and five from the thick-filament group). None of the patients experienced malignant arrhythmic events. When comparing the thin- and thick-filament groups, there were no differences in outcomes such as all-cause mortality, malignant arrhythmic events, or stroke. However, thin-filament HCM patients exhibited more rapid progression to advanced HF, with significance observed among probands (Figure 1). The predicted mean survival time until HF progression was 5.2 ± 0.64 years in the thin-filament group compared to 11.8 ± 1.04 years in the thick-filament group (*p* = 0.018). They also underwent septal reduction therapy less often. Only one patient (7%) in the thin-filament group had alcohol septal ablation, compared to twelve (17%) in the thick-filament group who underwent septal myectomy (*p* = 0.025). Nine patients in the thick-filament group (12%) and none in the thin-filament group received an ICD; the difference was not significant (*p* = 0.18).

### 3.3. Comparison of Thin-Filament HCM Patients Across Different Studies

Data on baseline characteristics and outcomes from our study, the adult studies by Coppini et al. (2014) and van Driest et al. (2003), and the pediatric study by Norrish et al. (2024) are shown in Table 2.

The predominant genotype in our cohort was *TPM1*, whereas in all other studies it was *TNNT2*. Other parameters in our group were in close agreement with the findings from adult cohorts reported by Coppini et al. and van Driest et al., including a low prevalence of thin-filament variants among genotyped patients (approximately 6%), age at diagnosis of 40–45 years, LV wall thickness < 20 mm, prevalence of LV outflow tract obstruction (LVOTO) at an average of 30%, and a significant prevalence of late gadolinium enhancement (> 80%). In our cohort, 20% of patients were relatives, whereas two other adult cohorts included only unrelated individuals. In contrast, the majority of the pediatric population (two-thirds) consisted of relatives. Compared with adult cohorts, pediatric patients predictably had a higher prevalence of familial HCM, lower LV wall thickness values, and a significantly lower incidence of LVOTO.

Septal reduction therapy was performed in a minority of thin-filament HCM patients in our cohort (7%) and in Coppini’s study (14%). This contrasts with the findings from Driest et al., who reported high rates of myectomy in both thin-filament (32%) and non-thin-filament (41%) groups, which they attributed to a surgical referral bias. In the pediatric population, septal reduction therapy was performed at the lowest rate (5%).

The follow-up period across studies was comparably averaged at nearly five years. In all four studies, major adverse events such as malignant arrhythmias, stroke, or death were rare or absent among thin-filament patients, with no significant differences compared to the thick-filament groups. The proportion of patients who developed severe HF symptoms was significant and comparable between our cohort (20%) and Coppini’s (15%). In addition, a notable 18% of patients in Coppini’s cohort completed the study with severe LV dysfunction, defined as an LV ejection fraction below 50%. In contrast, no pediatric patients experienced HF events. Interestingly, a substantially higher proportion of pediatric patients (62%) underwent ICD insertion compared to adult cohorts (0–24%).

## 4. Discussion

Studies on HCM associated with thin-filament gene variants are limited and mainly consist of case series or focus on individual genes [12,13,15,16]. Beyond our work, only three studies have analyzed thin-filament HCM as a group without gene preselection: Coppini et al. [17], van Driest et al. [20], and Norrish et al. [21]. Thin-filament variants are found in approximately 6% of genotyped adult HCM cohorts, a consistent finding across studies, including our own. This low prevalence makes the study of this subgroup challenging.

Our study, along with Coppini’s, demonstrated that LV wall thickness is lower in thin-filament compared to thick-filament HCM. In the pediatric cohort, baseline differences in LV wall thickness were not significant. Although van Driest et al. reported no statistical significances in any of the baseline characteristics, the mean LV wall thickness in the thin-filament group was below 20 mm, compared to over 21 mm in the rest of the cohort. These findings are consistent with earlier studies [12,16]. Collectively, this evidence indicates that in adults, the thin-filament genotype is strongly associated with the ‘thinner’ phenotype.

Septal reduction therapy was less common in the thin-filament group in both our and Coppini’s studies. Coppini et al. attributed this to the lower prevalence of LVOTO, whereas we associated it with a thinner septum, potentially affecting surgical decisions. The prevalence of LVOTO in thin-filament HCM ranged from 5% in the pediatric population to 42% in van Driest’s study. Notably, only Coppini et al. reported a significant difference in LVOTO prevalence between thin- and thick-filament HCM groups (19% vs. 34%). These findings suggest that the presence of LVOTO is unlikely to be directly influenced by genotype.

In our study and Coppini’s, patients with thin-filament HCM progressed more rapidly to advanced HF. Myocardial fibrosis, a key contributor to HF, was frequently observed on CMR in both- thin and thick-filament groups across both studies. While we did not assess fibrosis extent, Coppini’s study found significantly greater fibrous tissue in thin-filament patients, potentially explaining these findings. The pediatric study showed no difference in HF outcomes between thin- and thick-filament patients, likely due to the relatively short follow-up. In child-onset HCM, ventricular arrhythmias are the most common events during the first decade after baseline visit, while HF predominates by the end of the second decade of follow-up [22]. A recent meta-analysis by Saul et al., including 177 thin-filament HCM patients (two-thirds were relatives) from 21 studies, reported high HF morbidity and mortality, which is consistent with our conclusions [23]. In summary, our study supports the evidence that thin-filament HCM is characterized by a thinner phenotype and a worse prognosis for HF in adults. In some cases, this may delay diagnosis and calls for more careful monitoring of HF symptoms in these patients.

While early studies on *TNNT2*- and *TPM1*-associated HCM reported a high SCD risk [12,13], subsequent research has demonstrated that the overall arrhythmic risk in HCM caused by mutations in the *TNNT2* gene is comparable to that of the broader HCM population [16]. In our thin-filament group, the ‘thinner’ myocardium accounts for the lower 5-year SCD risk scores, as the other parameters in the calculator were similar to the thick-filament group. None of our thin-filament patients developed malignant arrhythmias or SCD during follow-up; none had a 5-year SCD risk score >6% and received an ICD. One subject had a family history of multiple SCD, which is an indication for an ICD according to American guidelines [24]. The rate of malignant arrhythmic events was similar between thin- and thick-filament groups, which is consistent with Coppini’s findings. In the pediatric study, children with thin-filament variants more frequently experienced NSVT and underwent ICD insertion compared to those with thick-filament variants. However, similar to adult studies, malignant arrhythmic events did not differ significantly. The authors suggested that the higher ICD insertion rate in thin-filament children might partially due to their older age at the last follow-up [21]. In contrast to our and Coppini’s findings, a meta-analysis by Saul et al. reported a trend towards an increased risk of SCD and ventricular arrhythmias in patients with thin-filament HCM [23]. One explanation may be that the meta-analysis comprised both pediatric and adult-onset cases. It is also important to note that most of the studies included were case reports with a potential bias towards severe phenotypes.

Controversies surrounding the arrhythmogenic potential of thin-filament mutations reflect the broader challenge of using genetics to predict SCD. Identifying high-risk HCM patients for SCD remains difficult [25], and ongoing efforts continue to improve prediction models [26], which currently offer modest predictive accuracy. Genetics shows promise as a novel risk factor, but its place in SCD risk stratification is not yet fully established. While the sarcomere-positive genotype was incorporated into the 2022 SCD prevention guidelines [27], it was excluded from the 2023 cardiomyopathy guidelines due to insufficient evidence supporting its independent prognostic value [28]. This complexity likely arises from additional contributors influencing the expression of disease-causing mutations. Some theories suggest that less malignant genetic variants may cluster within families or individual genomes, exacerbating the effects of primary mutations [29]. Further research is needed to integrate genetics into SCD predictive models in HCM.

Beyond risk stratification, several other areas of research could benefit from a focused study of thin-filament HCM.

Recent experiments have shown that while the first-in-class myosin inhibitor mavacamten is known to reverse the increased contractility caused by thick-filament mutations, its effect on HCM thin-filament mutations through a decrease in calcium sensitivity only partially rescues the contractile cellular phenotype and, in some cases, may exacerbate the effect of the mutation [30]. Therefore, new therapeutics should be investigated in relation to the genotype status of the patients, including whether they belong to thin- or thick-filament HCM.

The relationship between exercise and HCM has been extensively studied, and accumulating evidence supports the safety of vigorous exercise in HCM patients [31]. These findings have led to a significant reduction in previously strict exercise restrictions [32]. Research in murine models suggests that exercise may prevent or reverse fibrosis, myocyte disarray, and myocardial hypertrophy in HCM, though these effects have only been demonstrated in specific myosin mutations [33]. Despite these findings, rare but dramatic fatal events during sports still occur. A notable gap exists in studies investigating the impact of exercise on HCM progression with regard to genetic variations. Mutations in thin-filament genes associated with HCM increase myofilament Ca^2+^ sensitivity [11] and may potentially exacerbate exercise-induced morphological progression or arrhythmic events. Studies examining the role of physical activity in HCM patients with different genotypes are warranted.

The primary limitation of our study is the small sample size. Additionally, the five-year follow-up duration may be inadequate for a slowly progressive disease with a low event rate, such as HCM. Furthermore, a portion of the study group consisted of relatives, which may have contributed to a milder overall phenotype within the cohort.

## 5. Conclusions

Patients with thin-filament HCM face an elevated risk of rapid HF progression despite their ‘thinner’ phenotype. Evidence regarding a higher risk of malignant arrhythmias in thin-filament HCM remains inconsistent across studies and rather depends on additional factors such as age of onset and specific genotypes within individual families. The genetic profile of HCM patients should be considered in future prognostic and treatment response studies. 

## Figures and Tables

**Table 1:** Comparison of baseline characteristics between thin- and thick-filament HCM (all continuous variables shown as median (IQR)).

	Thin-Filament HCM n = 15	Thick-Filament HCM n = 67	*p*-Value
*Demography and Medical History*			
Age, years	49 (19)	45 (24)	0.35
Age at diagnosis, years	44 (20)	39 (22)	0.14
Probands, n (%)	12 (80)	57 (85)	0.44
Male sex, n (%)	6 (40)	37 (55)	0.29
Symptomatic, n (%)	8 (53)	44 (66)	0.37
**5-year SCD risk score, %**	**2.0 (1.8)**	**3.3 (2.9)**	**0.002**
5-year SCD risk score > 6%, n (%)	0	12 (20)	0.06
NSVT, n (%)	2 (15)	17 (30)	0.25
Family HCM in probands only, n (%)	7 (58)	26 (46)	0.42
Family SCD * in probands only, n (%)	2 (17)	6 (11)	0.42
NT-proBNP, pg/mL	810 (970)	845 (2083)	0.63
*Echocardiography*			
**Maximal LVWT, mm**	**17 (5)**	**21 (5)**	**0.024**
LVEF, %	71 (14)	67 (12)	0.60
LVOTO, n (%)	5 (33)	24 (36)	0.86
Rest LVOTO, n (%)	3 (20)	13 (19)	0.60
E/e’	9.5 (8.1)	8.9 (5.8)	0.76
LA diameter, mm	42 (13)	43 (11)	0.15
*Contrast Cardiac Magnetic Resonance*			
Number performed, n (%)	8 (53)	37 (55)	0.89
**Maximal LVWT, mm**	**17 (4)**	**21 (5)**	**0.006**
**Indexed LV EDV, mL/m^2^**	**61 (27)**	**70 (25)**	**0.041**
LGE, n (%)	7 (88)	32 (87)	0.71
*Electrocardiogram*			
QRS, msec	100 (18)	95 (23)	0.32
Sokolow–Lyon index, mm	25.5 (11)	25 (16)	0.85
Q waves, n (%)	3 (20)	15 (22)	0.57
T-wave inversion, (%)	8 (53)	42 (63)	0.50

HCM—hypertrophic cardiomyopathy; IQR—interquartile range; SCD—sudden cardiac death; NSVT—non-sustained ventricular tachycardia; NT-proBNP—N-terminal pro-brain natriuretic peptide; LVWT—left ventricular wall thickness; LVEF—left ventricular ejection fraction; LVOTO—left ventricular outflow tract obstruction; E/e’—early transmitral flow velocity to early mitral annular tissue velocity to estimate LV filling pressure; LA—left atrial; LV EDV—left ventricular end-diastolic volume; LGE—late gadolinium enhancement. * First-degree relatives under 40 years of age.

**Table 2:** Characteristics of thin-filament HCM across different studies (categorical variables shown as %).

	Our Group 2024 n = 15	Coppini et al., 2014 [17] n = 80	Driest et al., 2003 [20] n = 18	Norrish et al., 2024 [21] n = 21
Population	Adult	Adult	Adult	Pediatric
Prevalence in genotyped HCM	6.5	8	4.6	-
Probands	80	100	100	35
Genetics *				
*TPM1*	60	9	16	24
*TNNT2*	13	54	44	52
*TNNI3*	13	30	33	14
*TNNC1*	7	0	0	0
*ACTC1*	7	8	6	9.5
*Baseline*				
Age at diagnosis, years	45 ± 13	44 ± 16	40 ± 18	13 (6)
Male sex	40	55	61	71
Symptomatic	53	54	68	43
Family HCM *	58	44	39	71
Family SCD *	17	36	21	38
NSVT	13	30	-	16
Maximal LVWT, mm	17 (5)	18 ± 5	19.8 ± 6	15 (16)
LVEF, %	71 (14)	65 ± 10	-	-
LVOTO	33	19	42	5
LGE	88	85	-	75
Infero-lateral Q	20	37	-	-
T-wave inversion	53	67	-	-
Treatment **				
Beta blockers	40	67	-	-
ICD	0	24	11	62
Septal reduction therapy	7	14	32	5
*Follow-up*				
Duration, years	4.7 ± 2.3	4.7 ± 2.7	-	5.0 (4.5)
Type	Prospective	Prospective	-	Prospective
Death	0	2	-	0
Malignant arrhythmias ***	0	13	-	14
Progression to NYHA class III/IV	20	15	-	-
Dropped LVEF < 50%	0	13	-	0
Stroke	0	4	-	0

HCM—hypertrophic cardiomyopathy; SCD—sudden cardiac death; NSVT—non-sustained ventricular tachycardia; LVWT—left ventricular wall thickness; LVEF—left ventricular ejection fraction; LVOTO—left ventricular outflow tract obstruction; LGE—late gadolinium enhancement; ICD—implantable cardioverter–defibrillator; NYHA—New York Heart Association. * In probands only. ** Within a single time period, including past history and follow-up. *** Resuscitated cardiac arrest or appropriate ICD shocks.

## Data Availability

The data that support the findings of this study are available within the manuscript.

## Supplementary Materials

The following supporting information can be downloaded at https://www.mdpi.com/article/10.3390/jcm14030866/s1: Table S1: Sarcomeric gene variants identified in the study group.
