# Supplementary material for: Clinical Features and Prospective Outcomes of Thin-Filament Hypertrophic Cardiomyopathy: Intrinsic Data and Comparative Insights from Other Cohorts
Source: J Clin Med. 2025 Jan 28;14(3):866. doi: 10.3390/jcm14030866 (PMC11818361; doi:10.3390/jcm14030866)
Supplement: Supplementary file 1 [file jcm-14-00866-s001.zip › jcm-3425548-supplementary.pdf]

Table S1. Sarcomeric Gene Variants Identified in the Study Group.

Gene	Variant	Type	Novel^	Allele frequency*	Family segregation	Probands	Pathogenicity**
ACTC1	NM_005159.5:c.940C>T:p.R314C	missense		0		1	LP
TNNI3	NM_000363.5:c.557G>A:p.R186Q	missense		1.24e-6		1	P
TNNI3	NM_000363.5:c.422G>A:p.R141Q	missense		6.82e-6		1	P
TNNT2	NM_001276345.2:c.341C>T:p.A114V	missense		2.66e-5		1	P
TNNT2	NM_001276345.2:c.862C>T:p.R288C	missense		5.13e-4		1	LP
TPM1	NM_001018005.2:c.629A>G:p.Q210R	missense		6.20e-7	Y	3	LP
TPM1	NM_001018005.2:c.74C>T:p.A25V	missense		0	Y	1	LP
TPM1	NM_001018005.2:c.86A>G:p.K29R	missense		6.22e-7		1	VUS-LP
TPM1	NM_001018005.2:c.574G>A:p.E192K	missense		1.86e-6		1	P
MYBPC3	NM_000256.3:c.2965del:p.E989Sfs*3	frameshift	Y	0	Y	1	P
MYBPC3	NM_000256.3:c.2827C>T:p.R943*	stop codon		1.30e-5		2	P
MYBPC3	NM_000256.3:c.1351+2T>C	splice		6.20e-7	Y	1	P
MYBPC3	NM_000256.3:c.3697C>T:p.Q1233*	stop codon		8.67e-6	Y	9	P
MYBPC3	NM_000256.3:c.743_746del: p.D248Afs*51	frameshift		1.86e-6		1	P
MYBPC3	NM_000256.3:c.966G>A:p.W322*	stop codon		6.21e-7		1	P
MYBPC3	NM_000256.3:c.C2783A:p.S928*	stop codon		0		1	LP
MYBPC3	NM_000256.3:c.1037G>A:p.R346H	missense		4.96e-6	Y	3	LP
MYBPC3	NM_000256.3:c.927-9G>A	splice		1.21e-5	Y	1	LP
MYBPC3	NM_000256.3: c.3407_3409del:p.Y1136del	deletion		4.35e-6		1	LP
MYBPC3	NM_000256.3: c.2781_2782insCACA: p.S928Hfs*124	frameshift	Y	0	Y	1	P
MYBPC3	NM_000256.3:c.2738-1G>A	splice		0		1	P
MYBPC3	NM_000256.3:c.2345A>G:p.N782S	missense		5.15e-6	Y	1	VUS-LP
MYBPC3	NM_000256.3:c.2623C>T:p.H875Y	missense		0		1	VUS-LP
MYBPC3	NM_000256.3:c.3773T>A:p.L1258*	missense		6.20e-7		1	LP
MYBPC3	NM_000256.3:c.971del:p.I324Tfs*26	frameshift	Y	0		1	LP
MYBPC3	NM_000256.3:c.1731G>A:p.W577*	stop codon		6.21e-7		1	P
MYBPC3	NM_000256.3:c.1273C>T:p.Q425*	stop codon		0		1	P
MYBPC3	NM_000256.3:c.624G>C:p.Q208H	missense		1.30e-4		1	VUS#
MYBPC3	NM_000256.3:c.2441_2443del:p.K814del	deletion		3.72e-5		1	LP
MYBPC3	NM_000256.3:c.2429G>A:p.R810H	missense		7.19e-5		1	P

MYBPC3	NM_000256.3:c.1120C>T:p.Q374*	stop codon		0		1	P
MYBPC3	NM_000256.3:c.3811C>T:p.R1271*	stop codon		7.46e-6		1	P
MYH7	NM_000257.4:c.746G>A:p.R249Q	missense		6.20e-7	Y	1	P
MYH7	NM_000257.4:c.2156G>A:p.R719Q	missense		0		1	P
MYH7	NM_000257.4:c.2156G>A:p.R719W	missense		3.10e-6	Y	1	P
MYH7	NM_000257.4:c.1208G>A:p.R403Q	missense		6.19e-7	Y	1	P
MYH7	NM_000257.4:c.1283C>T:p.A428V	missense		0	Y	1	P
MYH7	NM_000257.4:c.T968C:p.I323T	missense		2.91e-5	Y	1	LP
MYH7	NM_000257.4:c.632C>T:p.P211L	missense		2.23e-5		1	LP
MYH7	NM_000257.4:c.2276G>A:p.G759D	missense		0		1	LP
MYH7	NM_000257.4:c.2129C>T:p.P710L	missense		0		1	LP
MYH7	NM_000257.4:c.2221G>A:p.G741R	missense		1.86e-6	Y	1	P
MYH7	NM_000257.4:c.2791G>A:p.E931K	missense		0	Y	1	P
MYH7	NM_000257.4:c.2185G>C: p.A729P	missense		0	Y	2	P
MYH7	NM_000257.4:c.5134C>T:p.R1712W	missense		6.20e-6		1	LP
MYH7	NM_000257.4:c.4163A>G:p.E1388G	missense	Y	0		1	LP
MYH7	NM_000257.4:c.5135G>A:p.R1712Q	missense		1.67e-5		1	P
MYH7	NM_000257.4:c.3652G>C:p.E1218Q	missense		6.20e-7		1	VUS-LP
MYH7	NM_000257.4:c.4180G>A:p.A1394T	missense		1.86e-6		1	VUS-LP
MYH7	NM_000257.4:c.1615A>G:p.M539V	missense		0		1	P
MYH7	NM_000257.4:c.3569C>T:p.A1190V	missense		1.88e-5		1	VUS-LP
MYH7	NM_000257.4:c.1711G>A:p.G571R	missense		3.72e-6		1	P
MYL2	NM_000432.4:c.275-8C>A	splice		6.20e-7		1	VUS-LP
MYL2	NM_000432.4:c.353+7G>A	splice		3.72e-6		1	VUS#
MYL2	NM_000432.4:c.49G>A:p.V17M	missense		9.29e-6		1	VUS-LP

N – no; Y – yes; P – pathogenic; LP – likely pathogenic; VUS – variant of uncertain significance; VUS-LP – VUS with high potential to be likely pathogenic; ACTC1 – alpha-actin; TNNI3 – cardiac troponin I; TNNT2 – cardiac troponin T; TPM1 – tropomyosin; MYBPC3 – cardiac myosin-binding protein C; MYH7 – myosin heavy chain; MYL2 – regulatory myosin light chain;

^Previously unreported

*The allele frequency of this variant in the gnomAD 4.1.0 database.

**Assessed using a) the predictive tool http://franklin.genoox.com, which integrates the ACMG classification, b) internal data such as family segregation, multiple unrelated variant carriers, absence of other suspicious variants, and c) analysis of the latest publications and ClinVar entries.

#Although pathogenicity of this variant remains uncertain, the variant was included in the analysis because the patient presented with a classical severe phenotype, and no other suspicious variants were identified when a comprehensive genetic panel was applied.
